# Tracking forest loss and fragmentation between 1930 and 2020 in Asian elephant (*Elephas maximus*) range in Nepal

**DOI:** 10.1038/s41598-021-98327-8

**Published:** 2021-09-30

**Authors:** Ashok Kumar Ram, Nabin Kumar Yadav, Pem Narayan Kandel, Samrat Mondol, Bivash Pandav, Lakshminarayanan Natarajan, Naresh Subedi, Dipanjan Naha, C. Sudhakar Reddy, Babu Ram Lamichhane

**Affiliations:** 1 Wildlife Institute of India (WII), Dehradun, India; 2 Ministry of Forests and Environment, Singhadurbar, Kathmandu, Nepal; 3 Ministry of Industry, Tourism, Forests and Environment, Province 2, Janakpur, Nepal; 4 National Trust for Nature Conservation (NTNC), Khumaltar, Lalitpur, Nepal; 5 Savannah Research Ecology Laboratory, University of Georgia, Athens, GA USA; 6 National Remote Sensing Centre, Indian Space Research Organization, Balanagar, Hyderabad, 500 037 India

**Keywords:** Biodiversity, Biogeography, Conservation biology

## Abstract

Forest cover is the primary determinant of elephant distribution, thus, understanding forest loss and fragmentation is crucial for elephant conservation. We assessed deforestation and patterns of forest fragmentation between 1930 and 2020 in Chure Terai Madhesh Lanscape (CTML) which covers the entire elephant range in Nepal. Forest cover maps and fragmentation matrices were generated using multi-source data (Topographic maps and Landsat satellite images of 1930, 1975, 2000, and 2020) and spatiotemporal change was quantified. At present, 19,069 km^2^ forest cover in CTML is available as the elephant habitat in Nepal. Overall, 21.5% of elephant habitat was lost between 1930 and 2020, with a larger (12.3%) forest cover loss between 1930 and 1975. Area of the large forests (Core 3) has decreased by 43.08% whereas smaller patches (Core 2, Core 1, edge and patch forests) has increased multifold between 1930 and 2020. The continued habitat loss and fragmentation probably fragmented elephant populations during the last century and made them insular with long-term ramifications for elephant conservation and human-elephant conflict. Given the substantial loss in forest cover and high levels of fragmentation, improving the resilience of elephant populations in Nepal would urgently require habitat and corridor restoration to enable the movement of elephants.

## Introduction

Deforestation and conversion of natural areas into human use impacts the earth's ecosystems and functions, and threatens biodiversity^1, 2^. The population of many wildlife species are declining globally, and about a million species are under threat of extinction primarily due to habitat loss/degradation, overexploitation, climate change, illegal wildlife trade, direct persecution, and conflict with humans^3–5^. Fragmentation is a significant factor leading to the loss of biodiversity in forested landscapes^6^. Habitat fragmentation affects ecological patterns and processes by increasing the number of forest patches, reducing the patch size, interrupting connectivity within the ecological network^7–9^, and impacting several species^10^. Habitat fragmentation could alter animal communities and trigger cascading effects on plants and ecosystem functions, including their carbon storage potential^11–13^. Continued fragmentation can lead to microclimatic changes in the edges, reduced core habitat, and eases the establishment of invasive species towards the forest interiors^14, 15^.

Effects of fragmentation on wide ranging large mammals like elephants is more severe and increases the extinction risks due to their needs for large and intact habitats^16–18^. With the current rise in anthropogenic impacts and loss of wildlife habitats, shared heterogenous landscapes around protected areas have immense potential for long term conservation of large mammals^19, 20^.

Elephants are the largest living terrestrial mammals facing typical threats of large mammals such as habitat loss, poaching and conflict with communities^21^. The increase in human population and expansion of agriculture had led to habitat loss and fragmentation, resulting in a significant decline in elephant populations across Asia and Africa^22–24^. Asian elephants are confined to 5% of the historic elephant range^24^. Elephants use large areas to meet their dietary and reproductive requirements^25, 26^. Their home range size varies according to the forage availability and nature of the habitat^27–29^. Expanding human settlements and agriculture areas has reduced connectivity, caused the loss of habitats, and a rise in human impacts on elephants, resulting in frequent conflict ranging from crop damage to human casualty and persecution of elephants^21, 30^.

Nepal is one of the elephant range countries, with an estimated 200 elephants in Nepal and additional 150 elephants seasonally migrating from India^31, 32^. The Chure Terai Madhesh Landscape (CTML) encompasses the entire elephant habitat in Nepal^33, 34^. Before the 1950s, the forest of CTML was reasonably intact^35^ and had a wide distribution of elephants that reportedly occurred in high densities^36–38^. A large tract of forests was converted into agriculture between 1950 and 1970, and wildlife was hunted down as agricultural pests with no legal protection^39^. Nearly three hundred wild elephants were also captured (287 between 1800 and 1975) and put in captivity for Royal hunting expeditions, forest patrolling, transportation and national security^37^. Deforestation led to the further decline of the elephant population.

In the CTML, the elephant ranges run east–west along the foothills of the Himalayas and Terai plains^33^. Protected areas (national parks and wildlife reserves) and forests outside the protected network constitute significant elephant habitats in CTML. The landscape includes both government and community-managed forests. Different landscape-level conservation approaches, including the Terai Arc Landscape (TAL) program, were implemented for biodiversity conservation in the CTML region^40^. However, CTML experienced significant habitat loss and fragmentation, contributing to the escalation of human-elephant conflict (HEC)^41^.

Despite increasing threats to the elephant conservation, landscape-specific information on the change in forest cover and habitat fragmentation is lacking for CTML and is urgently required for effective conservation planning for elephants. This study quantifies the change in forest cover for the last 90 years (1930–2020) using high-resolution imagery, estimating forest loss and habitat fragmentation in the CTML. We use the findings to relate habitat loss, fragmentation, and trends in HEC and discuss implications for elephant conservation in the human-dominated landscapes of Nepal and elsewhere. The findings will have implications for devising actionable strategies for elephant conservation and protecting existing forested habitats within human-dominated landscapes in Nepal.

## Results

### Temporal change of forest cover in CTML

We estimated 24,315 km^2^ of forest cover in 1930. The forest cover was reduced to 19,069 km^2^ in 2020, with an annual rate of 0.27%. The deforestation rate (0.29%) was higher between 1930 and 1975. The highest rate of deforestation was documented in western region (0.33%) followed by eastern (0.29%), far western (0.28%) and central (0.16%) region between 1930 and 2020 (Table 1). In 2020, the far western region had the highest forest area (35.42%), followed by the western region (26.18%), central (19.78%), and eastern region (18.61%) of CTML (Table 1).

**Table 1:** Status of forest cover by area in 1930, 1975, 2000 and 2020 in Chure Terai Madhesh Landscape (CTML), Nepal.

Region	Forest Area in different years (km^2^)				Percentage forest change (annual rate of forest change)			
	1930	1975	2000	2020	1930–1975	1975–2000	2000–2020	1930–2020
Eastern	4607.92	4084.94	3781.68	3548.48	− 11.35 (− 0.27)	− 7.42 (− 0.31)	6.57 (− 0.32)	22.99 (− 0.29)
Central	4336.84	4162.02	3917.77	3771.95	4.03 (− 0.09)	5.87 (− 0.24)	3.87 (− 0.19)	13.03 (− 0.16)
Western	6703.66	5590.97	5186.52	4993.85	16.60 (− 0.40)	7.23 (− 0.30)	3.86 (− 0.19)	25.51 (− 0.33)
Far western	8667.14	7482.98	7167.34	6754.86	13.66 (− 0.33)	4.22 (− 0.17)	6.11 (− 0.30)	22.06 (− 0.28)
Total	24,315.56	21,320.92	20,053.32	19,069.14	12.32 (− 0.29)	5.95 (− 0.25)	5.16 (− 0.25)	21.58 (− 0.27)

The total forest change percentage and annual rate of forest change (in parenthesis) is presented for four different time periods.

### Spatial change in forest cover

**Figure 1:**
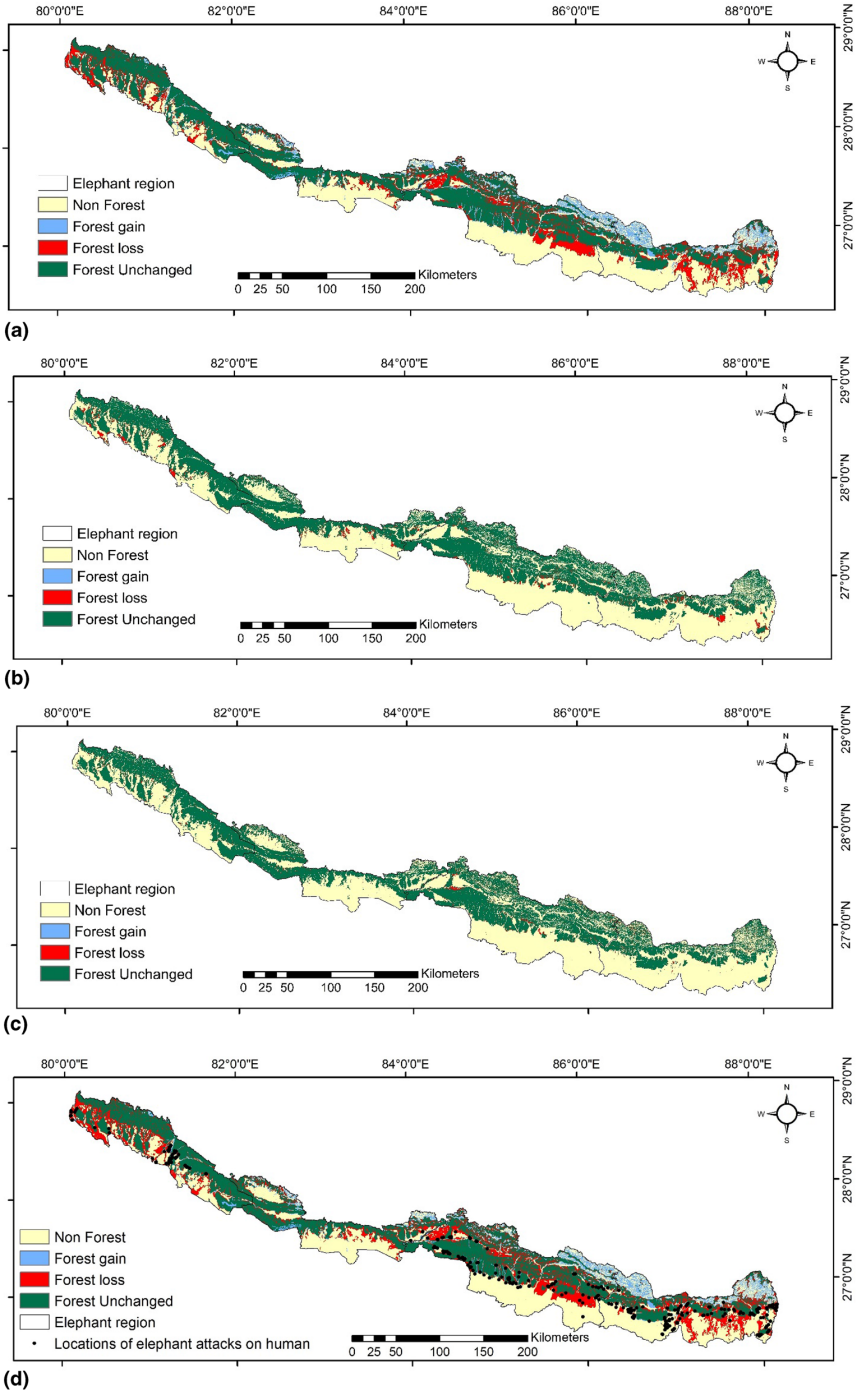
Forest cover change in the Asian elephant habitat (the Chure Terai Madhesh Landscape), Nepal during the time periods (**a**) 1930–1975, (**b**) 1975–2000, (**c**) 2000–2020 and (**d**) 1930–2020. In the map of (**d**) 1930–2020, locations of elephant attacks on humans based on Ram et al.^38^ is also overlaid. Map generated by Ashok Kumar Ram using ArcGIS 10.5^101^.

Altogether 1592 grids of 5 × 5 km^2^ were used to analyze the spatial patterns of forest cover change. Deforestation was documented in most of the grids (n = 1505), and 75 grids lost entire forest area between 1930–2020 (Supplementary figure S1). Increase in forest cover was observed in only 51 grids during the same period. The massive reduction in large forest patches (< 20 km^2^) was documented for 26 grids (Fig. 1a–c), (Supplementary table S4, Supplementary figure S2). We found ~ 60% of the elephant attacks on human (HEC) occurred in the area where forest was converted into the agriculture/settlements between 1930 and 2020 (Fig. 1d).

We calculated historical forest fragmentation for the last nine decades (1930–2020) and found that the total number of patches increased from 201 in 1930 to 28,559 in 2020. However, the highest decrease in mean patch size (121 km^2^ in 1930 decreased to 0.7 km^2^ in 2020) indicates that the forest has been fragmented into small patches. The mean perimeter ratio of the forest has been increased from 187 in 1930 to 1210 in 2020. The edge metrics showed that edge density increased from 548 (m/km^2^) to 3630 (m/km^2^), reduced mean patch edge to 2426.63 ha from 66,271.31 ha. Similarly, the shape index suggested that the mean shape index (MSI) decreased sharply where the mean perimeter area ratio (MPAR) increased progressively (Table 2).

**Table 2:** Forest fragmentation status in the CTML, Nepal in different time periods.

SN	Landscape metrics	1930	1975	2000	2020
1	**Patch density and size**				
a	Nump—no of patches	201	22,602	26,727	28,559
b	MPS—mean patch size (km^2^)	121	0.9	0.8	0.7
c	PSSD—patch size standard deviation	64,458.3	4643.1	3762.5	3187.2
2	**Edge metrics**				
d	ED—edge density (m/ km^2^)	548	2849	3263	3630
e	MPE—mean patch edge	66,271.3	2691.4	2451.4	2426.6
3	**Shape Index**				
f	MSI—mean shape index	1.8	1.4	1.4	1.4
g	MPAR—mean perimeter area ratio	187.4	1274.4	1245.5	1210.8
h	MPFD—mean patch fractal dimension	1.3	1.4	1.4	1.4

Between 1930 and 2020, 21.58% of the forest area was converted primarily into agriculture and settlements. The fragmentation analysis showed that the core forest (Core 3) size decreased by 43.08%, whereas, core 2 and core 1 size increased by 320.86% and 1107.33%. The patch area increased from 0.16 to 210.70 km^2^ between 1930 and 2020. The edge area increased from 1086.54 to 3269.72 km^2^, and the entire large core forests area (Core3) reduced significantly by 9968.68 km^2^ in 2020 (Table 3, Supplementary table S5).

**Table 3:** Temporal forest fragmentation (area in km^2^). Change percentage represented by ‘a’ means the highly inflated figure due to very small denominator.

Fragmentation class	1930	1975	2000	2020	Change 1930–1975	% Change (1930–1975)	Change 1975–2000	% Change (1975–2000)	Change 2000–2020	% Change (2000–2020)	Change 1930–2020	% Change (1930–2020)	% Change (1975–2020)
Patch	0.16	157.11	165.69	210.85	156.95	^a^	8.58	5.46	45.16	27.26	210.7	^a^	34.21
Edge	1086.55	2825.61	2910.84	3279.62	1739.06	160.05	85.23	3.02	368.78	12.67	2193.07	201.84	16.07
Perforated	0.67	1876.53	1430.23	1693.21	1875.86	^a^	− 446.3	− 23.78	262.97	18.39	1692.54	^a^	− 9.77
Core1	42.18	422.32	474.32	509.25	380.14	901.23	52	12.31	34.93	7.36	467.07	1107.33	20.58
Core2	49.34	157.51	182.84	207.65	108.17	219.23	25.34	16.08	24.81	13.57	158.31	320.86	31.83
Core3	23,136.67	15,881.84	14,889.4	13,168.56	− 7254.83	− 31.36	− 992.44	− 6.25	− 1720.84	− 11.56	− 9968.11	− 43.08	− 17.08
Total	24,315.56	21,320.92	20,053.32	19,069.14	− 2994.64	− 12.32	− 1267.6	− 5.95	− 984.19	− 4.91	− 5246.42	− 21.58	− 10.56

^a^The estimate is not reliable as forest cover within these categories were very small in 1930.

The Eastern region lost 22.99% of forest between 1930 and 2020. The core 3 decreased by 57.34%, whereas core 1 and core 2 increased simultaneously by 1019.26% and 409.08%. Similarly, the edge area increased by 219.91%. The central region lost 22.06% of the forest between 1930 and2020; core 3 decreased by 46.36%, whereas core 1 and core 2 increased by 4254% and 648%, respectively. The western region lost 13.03% of the forest, and core 3 forests were reduced by 30.88%, whereas core 1 and core 2 increased by 488% and 162%, respectively. Finally, the far western region lost 5.51% of the forest, and the core 3 forest was reduced by 37.11%, whereas core 1 and core 2 increased by 663% and 145%, respectively.

**Figure 2:**
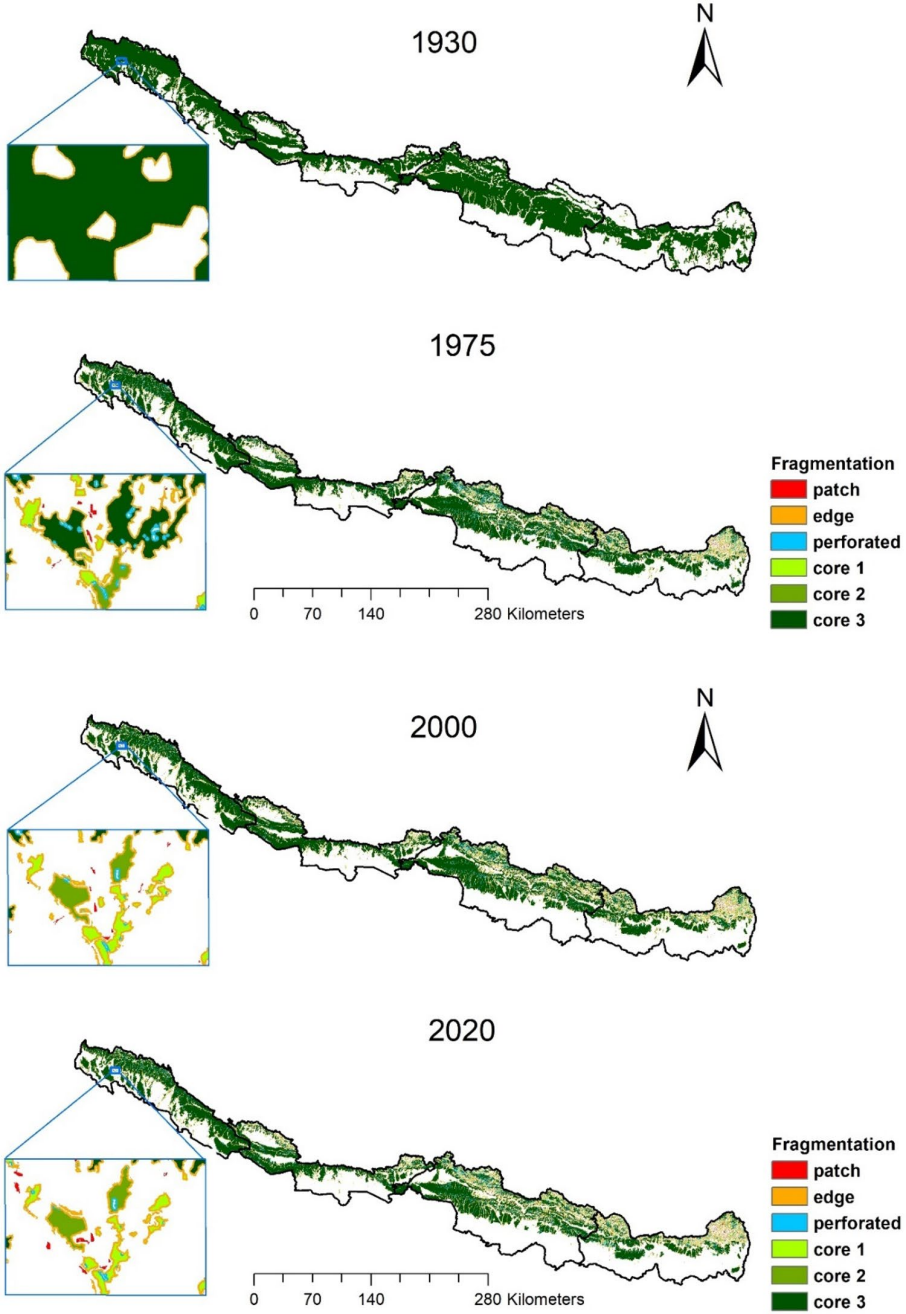
Habitat (forest) fragmentation of Asian elephants in the Chure Terai Madhesh Landscape, Nepal. Inset shows an enlarged view of habitat fragmentation in four different periods (1930, 1975, 2000 and 2020) at that particular location. Map generated by Ashok Kumar Ram using ArcGIS 10.5^101^.

The overall forest fragmentation result suggests that the highest fragmentation occurred in the eastern region (in core 3), followed by the central, far western, and western region, where the core forest (core 3) was reduced by 57.34%, 46.36%, 37.11%, and 30.88% simultaneously (Tables 3, 4, Fig. 2; Supplementary figure S2).

**Table 4:** Region wise forest fragmentation in Nepal. Change percentage represented by ‘a’ means the highly inflated figure due to very small denominator.

Fragmentation class (area in km^2^)	Eastern					Central					Western					Far western				
	1930	1975	2000	2020	1930–2020% change	1930	1975	2000	2020	1930–2020% change	1930	1975	2000	2020	1930–2020% change	1930	1975	2000	2020	1930–2020% change
Patch	0.01	69.68	333.42	93.36	− 933,500.00	0.14	49.51	224.21	75.1	− 53,542.86	0.000	22.21	192.38	23.4	^a^	0	15.71	215.33	18.99	a
Edge	297.17	863.34	1820.37	950.68	− 219.91	322.23	1011.77	2802.83	1252.36	− 288.65	219.920	463.55	1367.61	544.19	− 147.45	247.23	486.95	1726.4	532.38	− 115.34
Perforated	0.11	457.76	1360.58	423.75	− 385,127.27	0.11	826.08	3304.97	706.05	− 641,763.64	0.240	268.44	1649.26	271.11	− 112,862.50	0.21	324.25	2088.15	292.29	− 139,085.71
Core1	15.89	162.51	8.48	177.85	− 1019.26	4.28	133.75	22.79	186.38	− 4254.67	13.200	59.21	14.89	77.71	− 488.71	8.82	66.85	21.6	67.31	− 663.15
Core2	15.2	65.74	6.7	77.38	− 409.08	8.83	40.92	18.22	66.08	− 648.36	9.990	22.44	18	26.24	− 162.66	15.31	28.42	22.24	37.96	− 147.94
Core3	4279.55	2465.92	252.14	1825.46	57.34	8331.55	5420.95	794.33	4468.89	46.36	4093.490	3326.18	675.62	2829.29	30.88	6432.07	4668.8	1112.8	4044.91	37.11
Total	4607.92	4084.94	3781.68	3548.48	22.99	8667.14	7482.98	7167.34	6754.86	22.06	4336.840	4162.02	3917.77	3771.95	13.03	6703.66	5590.97	5186.52	4993.85	25.51

^a^The estimate is not reliable as forest cover within these categories were very small in 1930.

## Discussion

### Patterns of forest cover change and fragmentation

Our study provides comprehensive information on forest cover change and fragmentation within the primary elephant habitat in Nepal between 1930 and 2020. We documented the loss of more than one-fifth of the forest area and extensive fragmentation during this period. Our results suggest that the elephant habitat remained intact during the 1930s. However, the rate of deforestation was higher between 1930 and 1975 due to the conversion of forests into agricultural land. Forest cover loss was the highest in the western region, where the elephant population is the lowest. The regions with higher coverage of protected areas (central and far-western parts) had a comparatively lower rate of deforestation. Protected areas establishment (~ 6000 km^2^) and restoration through community-based conservation programs (~ 300 km^2^) may have contributed to reducing deforestation rates after 1975 in CTML^42^.

A previous study from the entire south Asia documented a 29.62% forest cover loss between 1930 and 2014 with a 0.68% annual rate of deforestation^43^.The forest loss and rate of deforestation in CTML are lower than the average for South Asia^14, 43^. ^44^also documented the annual rate of deforestation 0.49% for Nepal, which is higher than our results. Forests occupied 42.73% of CTML in 2020, but forest cover was not evenly distributed throughout the landscape. A large part of the remaining forest occurs in the Chure region (> 70% forested), where the rate of deforestation was comparatively lower (0.18%/year between 1995 and 2010)^42, 45^. However, most of the flat and productive land of the CTML was converted into agricultural land with a higher rate of deforestation (i.e., 0.40%/year) between 1991 and 2010^42^. Among the four regions, the western part experienced the highest loss of forest cover (25.51%). The remaining forest cover (56%) and rate of deforestation (0.33) were found higher in the western region, where almost the entire forests lie outside of the protected areas. Despite massive forest clearance in Chitwan valley^46^ and other areas of central CTML, the rate of forest loss was only 0.16% per year. The establishment of Chitwan and Parsa National Parks and the intact forest remaining in the northern part of Bara and Rautahat may have contributed to lower deforestation rates in Central CTML^47^. The results indicate that Government should prioritize conservation efforts to restore elephants' movement through corridor restorations within the human-dominated landscapes outside protected areas.

Elephant habitat is more fragmented outside protected areas due to the high pressure of encroachment and developmental activities^48^. These forests are also used more frequently by the local communities to meet their subsistence needs of livestock grazing and dependence on forest products^49, 50^. With increasing forest fragmentation, the elephants and other wildlife are also forced to live in smaller forest patches with spatial overlap with human activities^51^. This situation increases the chances of confrontation between humans and elephants, often leading to fatal attacks^34, 52^.The eastern region had the highest forest fragmentation (57.3% of large core forest lost) within our study, where HEC incidents were also the highest^34^(DNPWC 2020). The eastern region also bears a long migratory route of a large herd of elephants (> 100) and provides habitat for some residential elephants. Although the forest cover is not significant within Koshitappu Wildlife Reserve (KTWR), it still provides refugia and a corridor for elephants in the eastern region. While navigating through the highly fragmented forests, there is always a threat of elephants getting deflected due to haphazard drives and another form of human resistance resulting in elephants ending up in human-use areas off the forests, as corroborated by telemetry studies on elephants in the landscape.

Forest fragmentation results suggested that large forest patches have decreased rapidly, whereas forests in the medium and small core have increased massively. Similarly, the area of forests in the patch, perforated, and edge category has also increased during the last nine decades (1930–2020), which indicated the high rate of forest fragmentation in the CTML. Landscape metrics analysis also reveals the massive fragmentation of forests between 1930 and 2020, increasing the patch number and decreasing patch size (Table 3, 4; Fig. 2)^44^. Similar to Nepal, a massive decline in extensive core forests and increase in fragmented patches has been documented in other elephant range countries India, Myanmar, Bangladesh, and Sri Lanka^53–56^. Fragmented forest patches should be connected through a combination of the weak and high-quality habitat to enable elephant connectivity throughout the landscape. The human pressure (illegal cattle grazing, resources extraction) and risks of invasive species (*Lantana camera*, *Chromolaena odorata*, *Parthenium hysterophorus*, *Mikania micrantha*, etc.) spread are high in smaller and perforated forest patches as well as forest edges^57^.

Our study focused on forest cover change within elephant range. Elephants are habitat generalists and roam across large areas which comprises of a matrix of forests, grasslands, wetlands and agriculture areas^58^. Size of protected areas in Nepal are not large enough to sustain elephants throughout the year. The fragmented forest patches serve as refuge whereas crop fields around human settlements and periphery of protected areas act as attractants for elephants. Human-elephant conflict (HEC) intensifies along the periphery of these protected areas, forest patches. The ecotone habitats along the forest-agriculture matrix have high activity of humans leading to increased probability of human-elephant conflict^34^. Out of 412 cases of elephant attacks on humans (HEC), 60% occurred at forest cover loss areas during 2000–2020 in Nepal^34^ (Fig. 1d). Human activity around riverine patches, water bodies also reduce access to such important resources for elephants. Apart from strengthening forest patches, wildlife corridors it is also important to grow unpalatable crops within ecotone habitats to reduce visitation by elephants. These changes in land use patterns have to be integrated with necessary management interventions to reduce human-elephant conflicts^9, 59^. Large herbivores exhibit different strategies of habitat use with seasonal variation and spatial distribution of resources^60^. Studies on on habitat utilization pattern by elephants^61, 62^ shows that distribution of water resources^63, 64^, precipitation patterns^65^ and social factors^20^ influence their movement and spatial distribution. The intensity of conflict varies with distribution of resources, agricultural practices, human population, climatic conditions and connectively between habitats^66–69^.

### Drivers of deforestation and fragmentation

Several studies indicate loss of elephant habitats and fragmentation due to a combination of multiple factors such as agriculture and settlement expansion, encroachments, irrigation, infrastructure development hydropower projects, illegal logging, mining, commercial plantations^53, 61, 70, 71^. Additionally, expansion of oil palm plantation in Indonesia^72^ and tea, paddy cultivation in north-east India has also contributed to habitat loss^73^. However, in Nepal, forest conversion into farmlands through government policy was responsible for forest loss and fragmentation in the initial years (1930–1975), whereas encroachment and infrastructure development activities have continued the fragmentation at present and recent past and expansion of agriculture is a significant factor for conversion of elephant habitat in Nepal.

A large part of the forest was lost or fragmented in CTML during the first 45 years (1930–1975). During this period, various socio-political changes and national policy of promoting forest conversion into agricultural land in Terai have contributed to such massive fragmentation of the forests in CTML^39^. Three significant changes were (a) fall of Rana regime and political instability, (b) private forests nationalization act 1957 and its impacts, (c) land resettlement policy. Rana rulers used to grant forest and other lands as 'Birta' (grant their families and close relatives as private property) and provide to government employees and other servicemen to use a share of a product as a 'Jagir.' As a result, the tiny forest remained under government control^74^. After the fall of the Rana Regime in 1951, state of political instability in the country caused massive deforestation and wildlife hunting^75^. In the meantime, the government of Nepal nationalized all the private forests by promulgating the "Private Forest nationalization Act, 1957"^76^. As a result, owners of the private forests converted their forested land into farmlands to secure their land tenure^77, 78^. Similarly, eradicating malaria in CTML during the 1950s and introducing a new settlement policy by the government promoted thousands of hill migrants to convert Terai forests into farmlands^46^. The human population in the CTML also increased many folds during this period, accelerating deforestation and forest degradation^79^.

The deforestation rate was lower between 1975 and 2020. The primary reasons were ((a) establishment of protected area network, ((b) initiation of community participation in forest management, (c) well-established institutional setup for forest protection and management^74^. With decreasing deforestation and increasing forests and wildlife conservation efforts, wildlife populations, including the elephants, have also increased (~ 50 individuals in the 1970s to > 200 in 2020; Shrestha et al.^37,80^). However, the forest fragmentation continues in large parts of the forests outside of the protected areas. Large-ranging species like elephants are affected by this as they come into frequent clashes with humans while navigating seasonally through these highly fragmented forests in CTML.

This study indicates that the conservation of large-ranging species like elephants and tigers in CTML has been challenging as most of the remaining forests are highly fragmented, especially outside the protected areas. With planned and ongoing infrastructure development activities in CTML, forest fragmentation continues to increase. It shows the importance of the landscape-level conservation approach and helps policymakers, protected area managers to restore corridor and connectivity by implementing metapopulation management of large mammals in Nepal and around the globe.

## Conclusion

Forest loss and fragmentation induced a severe threat to elephant conservation in Nepal. Such fragmentation brought both the elephants and humans along the forest's edge, where they interact with each other, often resulting in severe human-elephant conflict (HEC). Increasing the number of forest patches also increases the visibility of elephants in the migratory routes, increasing the poaching threats. Our research findings have implications for devising appropriate policies for conserving large mammals and their habitats in human-dominated landscapes in Nepal and beyond. Further understanding of the relationship between forest loss/fragmentation and human-elephant conflict is necessary. The particular focus of elephant conservation is necessary outside the protected areas and migration corridors where habitat is highly fragmented.

## Methods and materials

### Study area

**Figure 3:**
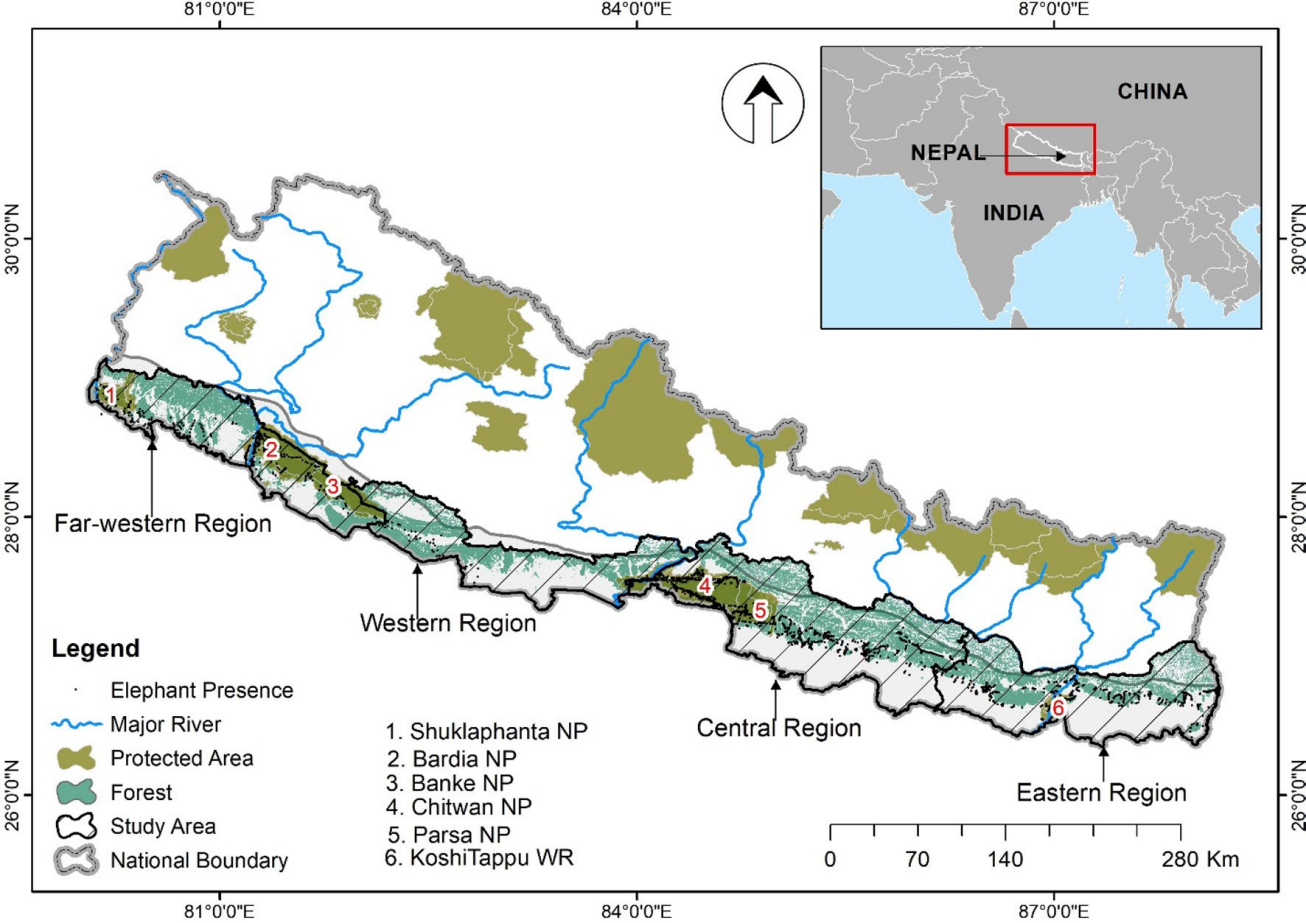
The geographical location of Nepal and the study area (Chure Terai Madhesh Landscape). Map generated by Ashok Kumar Ram using ArcGIS 10.5^101^.

Chure-Terai-Madhesh landscape (CTML) covers the entire elephant distribution range in Nepal. The CTML spreads across 25 districts and covers an area of 42,456 km^2^ (Fig. 3). CTML comprises five physiographic units i.e., Chure hills (34.4%); Chure narrow gorges (2.2%); Dun/Inner Tarai (8.4%); Bhavar region (14.9%); and Tarai Madhesh (40%). Forty-eight percent of the landscape comprises agriculture and settlement; 47.16% forest, shrub-land, and grassland; and the rest 4.65% river and riverbed^81^. CMTL is a part of the global biodiversity hotspot^82^ and provides essential environmental services such as groundwater recharge for more than half of Nepal's human population (~ 15 million)^83, 84^. The major habitat types are (a) Himalayan subtropical broadleaved forests, (b) Gangetic plains and moist deciduous forest, and (c) Terai-Duar savannas and grassland. Apart from elephants, the study area is also a refuge for several endangered large mammals, including the tiger (*Panthera tigris*), greater one-horned rhinoceros (*Rhinoceros unicornis*), Gaur (*Bos gaurus*), and wild buffalo (*Bubalus bubalis arnee*). The annual rainfall ranges between 1138 mm and 2680 mm, with over 80% of the rain occurring during monsoon months^42^. The altitudinal range lies between 60 and 1500 m^85^. CTML is densely populated with an average human density of 392 persons/km^2^^83^. Sixty percent of the people depend on subsistence agriculture and are involved in farm and off-farm based livelihood activities (Chaudhary and Subedi, 2019). Paddy (*Oryza sativa*), maize (*Zea mays*), wheat (*Triticum aestivum*), lentils (*Lens culinaris)* are some major food crops, where jackfruit (*Artocarpus heterophyllus*), mangoes (*Mangifera indica*), bananas (*Musa acuminata*) are some fruit crops farmed in the area^86^. Large-scale linear infrastructure projects and mining activities are the major drivers of deforestation and habitat fragmentation in the landscape.

We divided CTML into four regions (Eastern, Central, Western and Far-western) of similar size to assess the extent of forest loss (Table 5). Thus, elephants are distributed in four population clusters with limited connectivity viz. (a) eastern population (Mechi River to Kamala River), (b) central population (Kamala River to Narayani River), (c) western population (Narayani River to Western boundary of Dang district), and (d) far-western population (Eastern boundary of Banke district to Mahakali River)^87, 88^ (Table 5).

**Table 5:** Four different regions within Chure Terai Madhesh Landscape Nepal, area, forest cover and elephant population status.

SN	Region	Coverage (districts)	Total area (km^2^)	% forest cover	Elephant population
1	Eastern	Jhapa to Siraha	11,116.96	31.92	Residential: 27–35; ~ 100 migratory elephants each year from West Bengal, eastern India)
2	Central	Dhanusa to Chitwan	8169.43	46.17	Residential: 45–53
3	Western	Nawalparasi to Dang	8777.95	56.89	Migratory: 8–12
4	Far-Western	Banke to Kanchanpur	14,391.54	46.94	Residential: 80–125; ~ 45 migratory (from Uttarakhand and Uttar Pradesh, India migrated to far western habitats in Nepal)
	Total		42,455.88	44.92	

The elephant population was obtained from Ram and Acharya^80^.

### Derivation of forest cover

We analyzed forest cover change and fragmentation using both the patch and landscape metrics and considered forest fragmentation as habitat fragmentation^89^. Natural Forests or plantations covering greater than 0.5 ha area were categorized as forest^90^. We used the hybrid classification techniques to combine high-resolution images, medium resolution images, and digitization of topographic maps. First of all, we prepared a forest cover map of the 1930s by digitizing greenwash areas shown on topographical maps prepared by Army Map Service, U.S. Army, Washington, surveyed during 1920–1940 (http://legacy.lib.utexas.edu/maps/ams/india/) at 1:250,000 scale. Due to the unavailability of multi-spectral satellite images of the study area before the 1970s, we relied on the existing topographic maps to obtain forest cover of 1930^91, 92^.

Kaim et al*.*^93, 94^ found 5–10% inherent errors at various stages of land cover change analysis; while using historical data and topographic maps. The inaccuracy of forest cover mapping was minimized by visual interpretation and overlay analysis in the topographic maps. In addition, we resampled all the digital images at a 30-m resolution to improve the mapping errors. ^93, 94^reported the reliability of topographical maps to reconstruct forest cover. We also obtained the forest cover map of 1975 by on-screen digitization of Landsat 1 TM level 1 satellite images.

**Figure 4:**
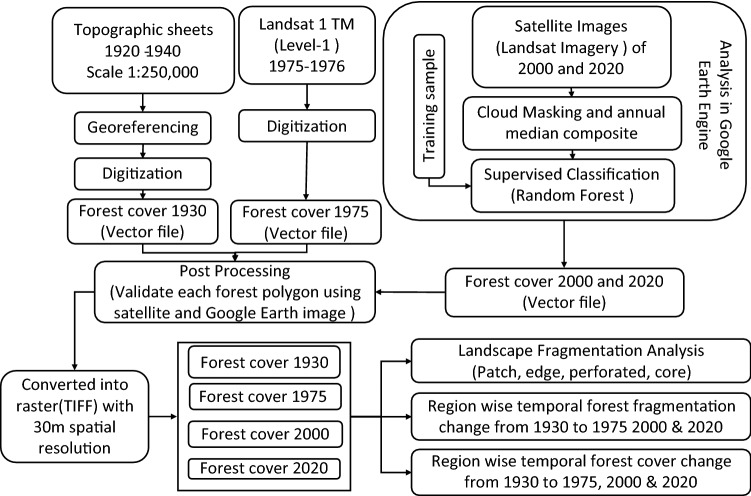
Overall methodological framework adopted for this study.

We produced the forest cover maps of 2000 and 2020 from Landsat imagery scenes respective years with < 10% cloud cover (Table 6; Fig. 4). All the Landsat data processing was conducted using the cloud-computing technology in the Google Earth Engine (GEE) platform (https://earthengine.google.org/). The GEE platform carried out a fast analysis using Google’s computing infrastructure^95, 96^. We used the pre-processed Landsat imagery available through GEE to assess forest cover change across the study area^97^.

**Table 6:** Sources of data used in this study.

SN	Data layer	Source	Spatial resolution (m)	Year
1	Topographic map	Army Map Service, U.S. Army, Washington	Scale 1:250,000 m (based on Arial photo)	1920–1940
2	Landsat 1 TM	Earth Explorer (USGS)	60 m	1975–1976
3	Landsat 5 Surface Reflectance Tier 1	GEE dataset (USGS)	30 m	2000
4	Landsat 8 Surface Reflectance GEE dataset	GEE dataset (USGS)	30 m	2020
5	Administrative boundary	Department of Survey, Nepal	Scale 1:25,000 (based on Arial photo)	1996–1998

We used a cloud screening algorithm to remove cloud contaminated pixels from each Landsat image by applying quality assessment (QA) bands for 2000 and 2020. Then, we produced an annual composite by taking the median value from images from the target year^98^. We delineated > 1000 reference points for each period 2000 and 2020, respectively. We used supervised machine learning classifiers, i.e., Random Forest (RF), to classify remotely sensed data^99^. Random Forest Classifier creates a set of decision trees from a randomly selected subset of the training set and aggregates the votes from different decision trees to classify the image^100^. The classified image was downloaded as raster tiff files. The raster was converted into vector polygons and overlaid with high-resolution google earth images of respective years. The final forest cover map was obtained with the highest accuracy by post-processing (validating) the forest polygons through onscreen digitization to match the forests visible in Google images^99^.

### Data analysis

#### Analysis of forest loss/gain

Forest cover maps of the four different periods of 1930 (before malaria eradication), 1975 (the initial stage of PA system development), 2000 (well-established PA system) and 2020 (current scenario) were post-processed according to FAO forest definition. These layers were analyzed to understand changes in extent and location of forests using a post-classification change detection technique in Arc GIS 10.5.

We estimated the conversion of forests into the non-forest area on a grid overlay basis. We generated 5 × 5 km^2^ grids for forest cover change analysis following Padaliya et al.^91^ and Reddy et al.^43^ for the time series assessment and analyzed spatial distribution trends of forest cover in these grids from 1930 to 1975, 1975 to 2000, and 2000 to 2020^53, 91^. We computed the forest cover area (distribution of transitions and persistence of forest) of four different periods in each grid using the zonal statistics tool of ArcGIS software^101^. Overall, forest cover change was calculated by combining all the grids and calculating the annual deforestation rate (percentage) using a compound-interest-rate formula^54^.

$$r=\frac{1}{t2-t1}\times ln\frac{\mathrm{a}2}{a1},$$

where a1 and a2 are the area covered by forest at times t1 and t2. The region wise rate of deforestation was computed and presented.

We also overlaid the Human elephant conflict (HEC) locations (Locations of elephant attacks on humans) of last 20 years (between 2000 and 2020) over the forest cover map of 1930 and 2020 (Fig. 1d) to examine the relation of HEC with forest cover change. We took HEC data (elephant attacks on humans) from the published article Ram et al.^34^.

#### Modeling forest fragmentation

We carried out habitat fragmentation analysis in the four regions of CTML (Fig. 3) and measured fragmentation in terms of core, perforated, edge, and patches. We used 30 m cell resolution for fragmentation analysis for four different periods. We used patch analyst^102^ to obtain the patch matrix for each region viz. patch density and size (number of patches, mean patch sizes, patch size standard deviation), edge metrics (edge density, mean patch edge), and shape index (mean shape Index, mean perimeter area ratio, mean patch fractal dimension) (Supplementary table S3).

Similarly, Landscape Fragmentation Tool (LFT V2.0, http://clear.uconn.Edu/tools/lft/lft2/) was used to estimate landscape metrics^103^. The change of fragmentation during the 1930–2020 periods was carried out by cross-tabulating the fragmentation classes. Landscape Fragmentation Tool (LFT) classifies forests at pixel-level into fragmentation classes: core 1, core 2, core 3, perforated, edge, and patch. Core forests are located far from the forest/non-forest boundary and surrounded by other forest areas. We considered the core forest as 100 m distance from the edge^104^. The core forests include three different types (1) Core 1: forest patches area < 250 acres (1.012 km^2^), (2) Core 2: medium core (forest patches area between 250 and 500 acres (1.01–2.2 km^2^), and Core 3: large core (forest patches area > 500 acres (> 2.2 km^2^)^91^. The peripheral forest was further classified into perforated (1) inner edge: forest pixels on the edge of small interior non-forest, and (2) edge forest or outer edge: pixels that are between forest and large non-forest areas^105^.

### Ethics approval

We obtained research permission from the Department of National Parks and Wildlife Conservation Nepal (Ref no: 3066/073/74; June 02, 2017). We did not carry out any experiments with live animals. We properly acknowledged the sources of data and supporting organizations/individuals for this research.

## Supplementary Information

Supplementary Information.

## Data Availability

Upon publication of the article, all the supporting data for obtaining the results will be made available via the online data services such as dryad.
