# Supplementary material for: Tracking forest loss and fragmentation between 1930 and 2020 in Asian elephant (Elephas maximus) range in Nepal
Source: Sci Rep. 2021 Sep 30;11:19514. doi: 10.1038/s41598-021-98327-8 (PMC8484620; doi:10.1038/s41598-021-98327-8)
Supplement: Supplementary file 1 — Supplementary Information. [file 41598_2021_98327_MOESM1_ESM.docx]

# **Tracking forest cover loss and fragmentation (1930–2020) in Asian elephant (*Elephas maximus*) habitats in Nepal**

Ashok Kumar Ram*^1,2^, Nabin Kumar Yadav^2^, Pem Narayan Kandel^2^, Samrat Mondol^1^, Bivash Pandav^1^, Lakshminarayanan Natarajan^1^, Naresh Subedi^4^, Dipanjan Naha^6^, C. Sudhakar Reddy^5^, Babu Ram Lamichhane*^4^

^1^ Wildlife Institute of India (WII), Dehradun, India

^2^ Ministry of Forests and Environment, Singhadurbar, Kathmandu, Nepal

^3^ Ministry of Industry, Tourism, Forest and Environment, Province 2, Janakpur, Nepal

^4^ National Trust for Nature Conservation (NTNC), Khumaltar, Lalitpur, Nepal

^5^ National Remote Sensing Centre, Indian Space Research Organisation, Balanagar, Hyderabad 500 037, India

^6^ Savannah Research Ecology Laboratory, University of Georgia, Athens, GA, USA

* Corresponding Authors Email: elephas.maximus@yahoo.com & baburaml@gmail.com

**Supplementary information**

*Supplementary figure* ***S1*.** Grid wise forest cover change (5x5 km^2^) a) 1930–1975, b) 1975—2000, c) 2000–2020


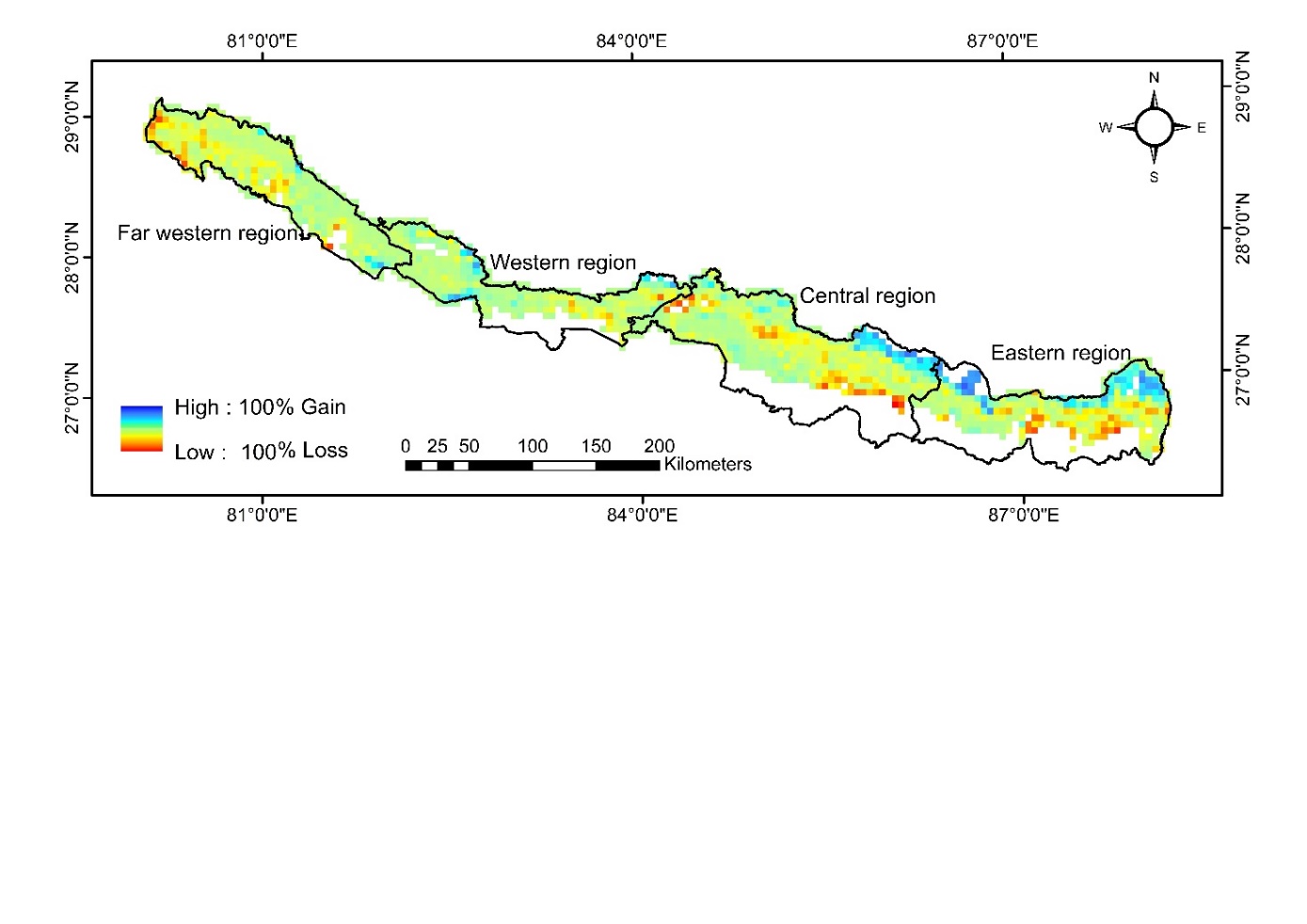
a)


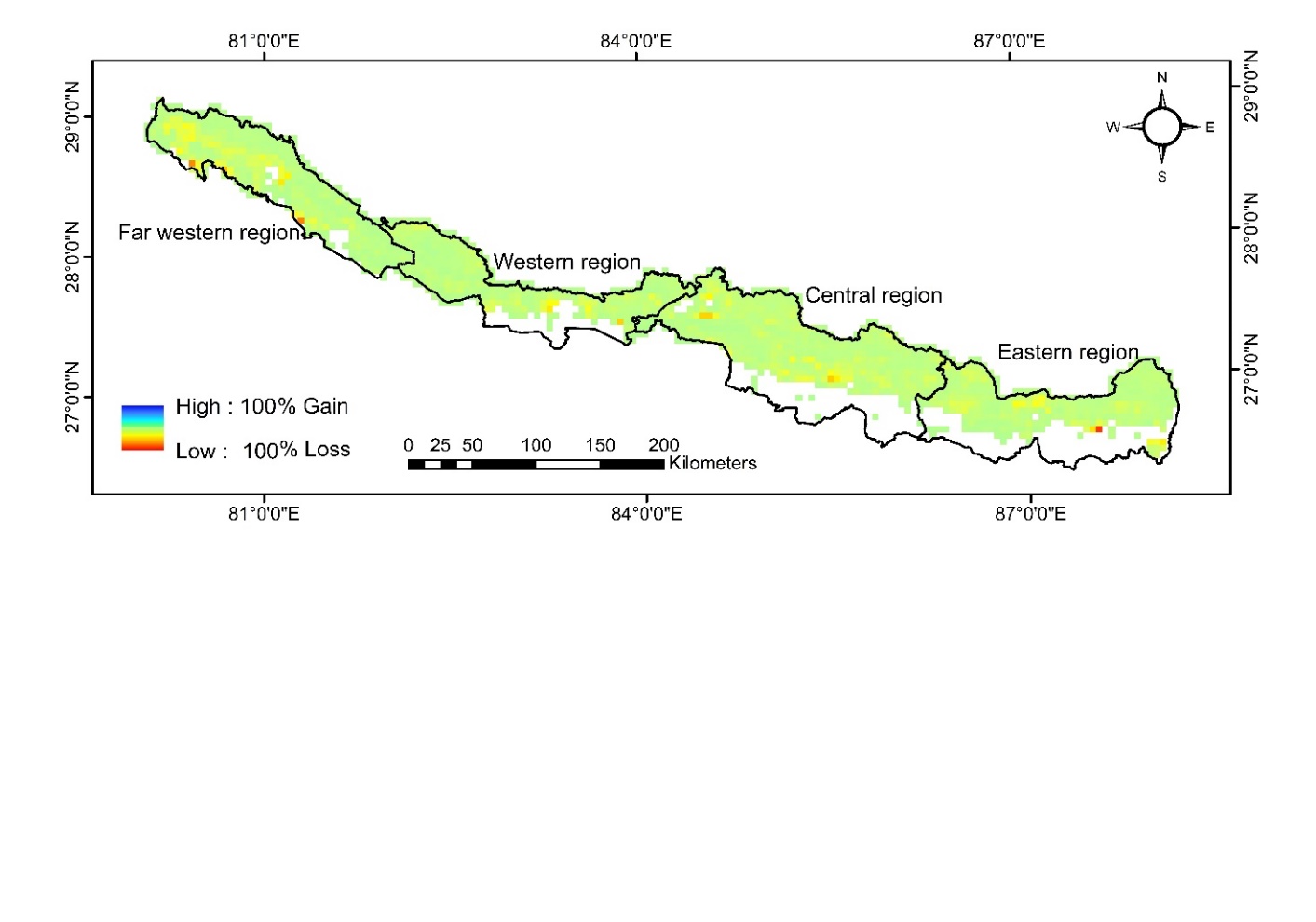
b)


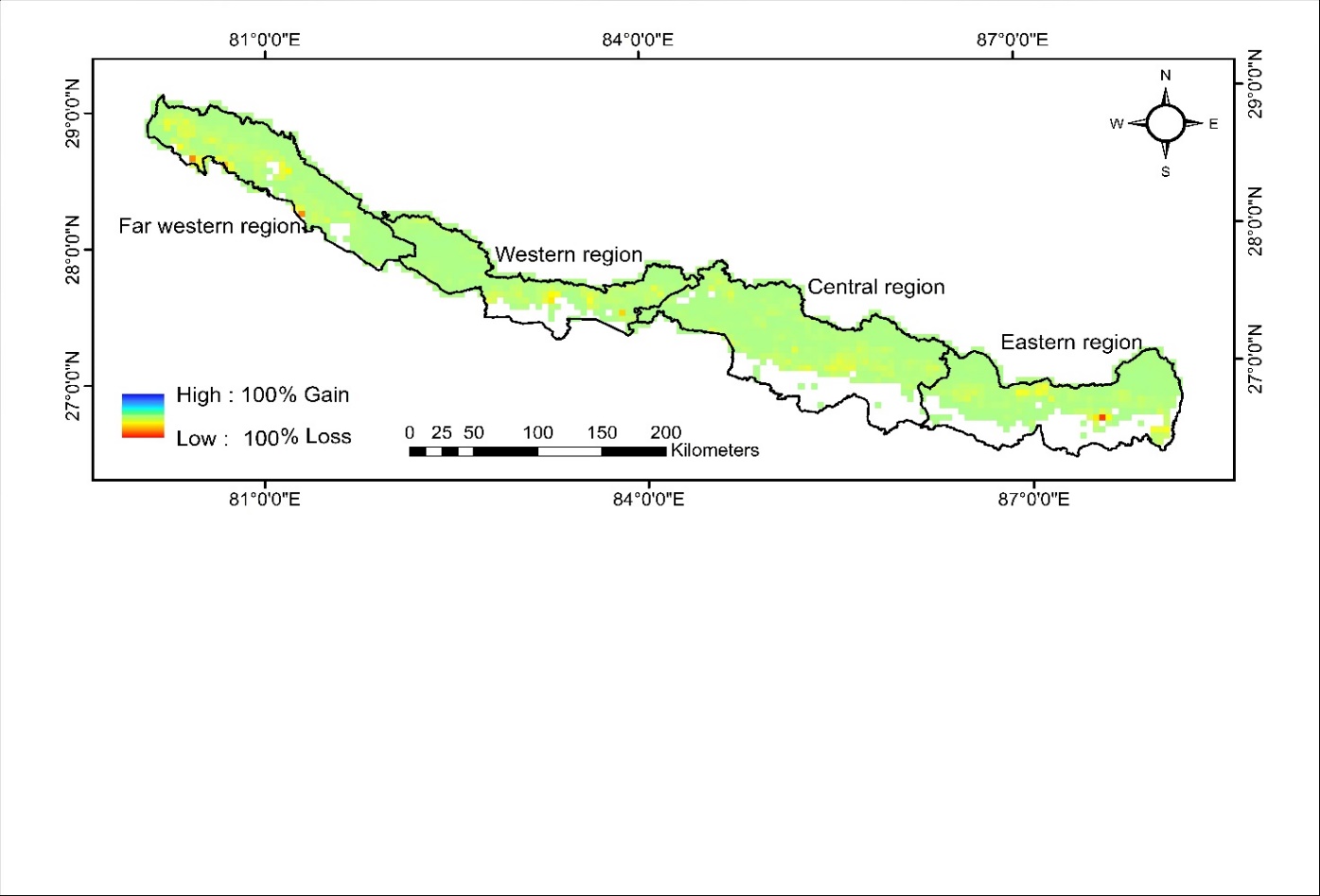
c)

*Supplementary figure* ***S2****.* Region wise forest fragmentation.


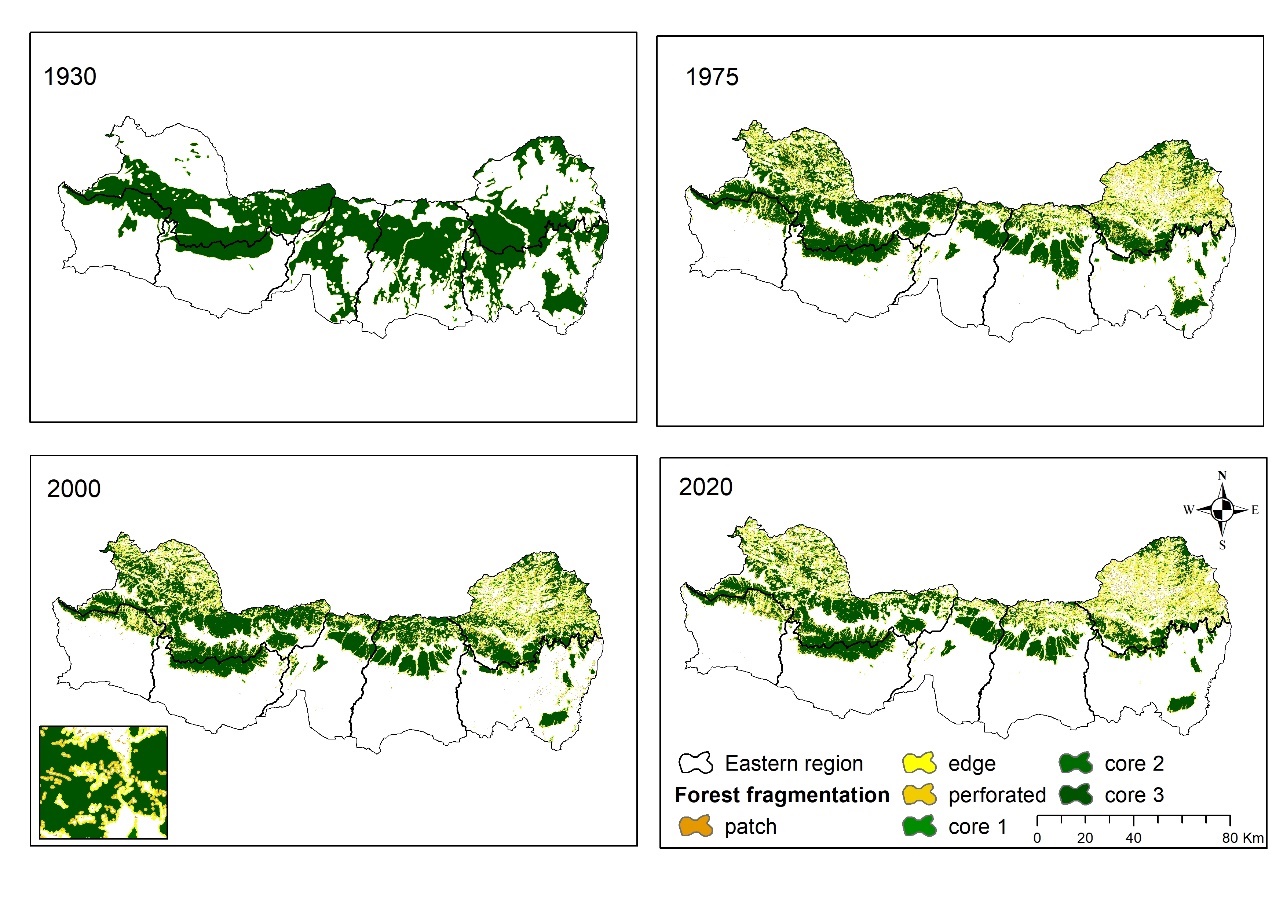


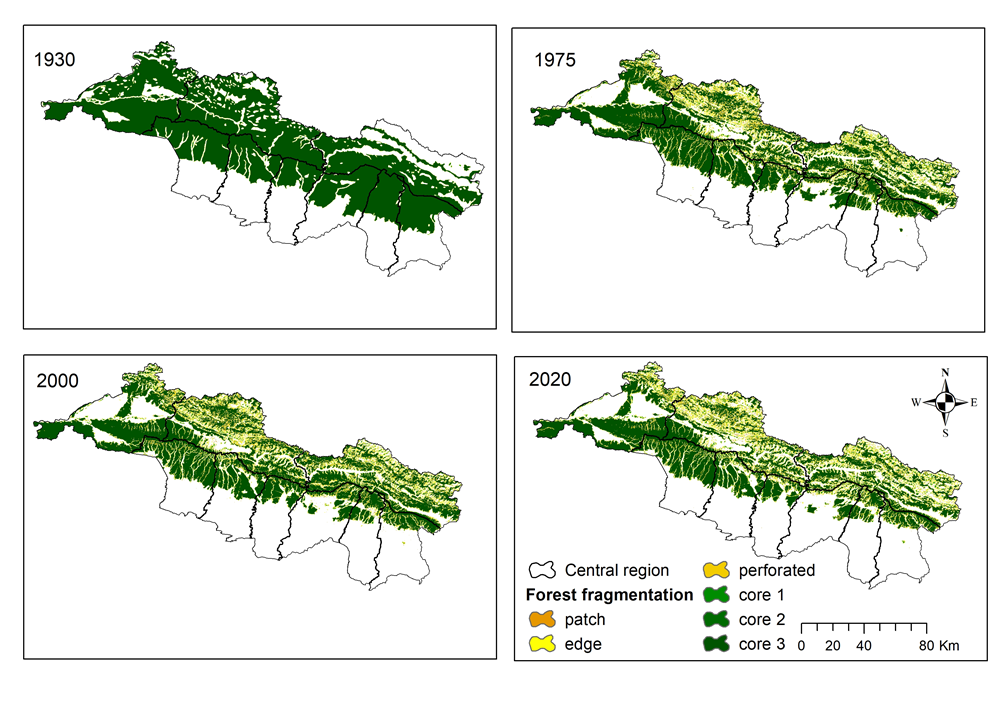


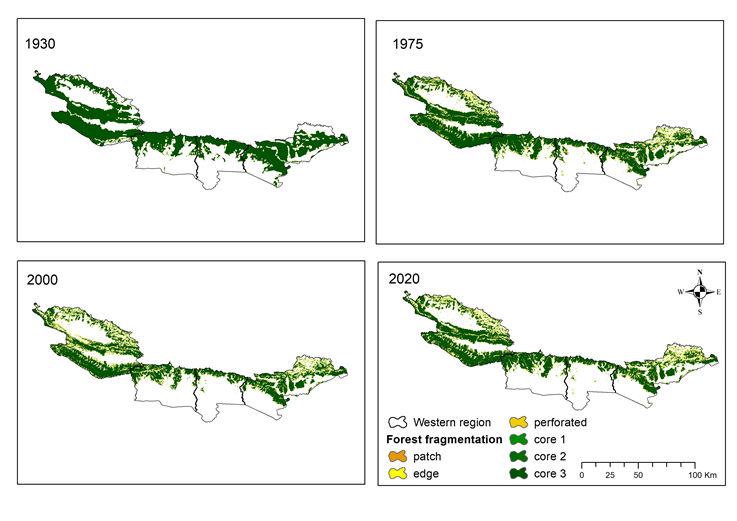


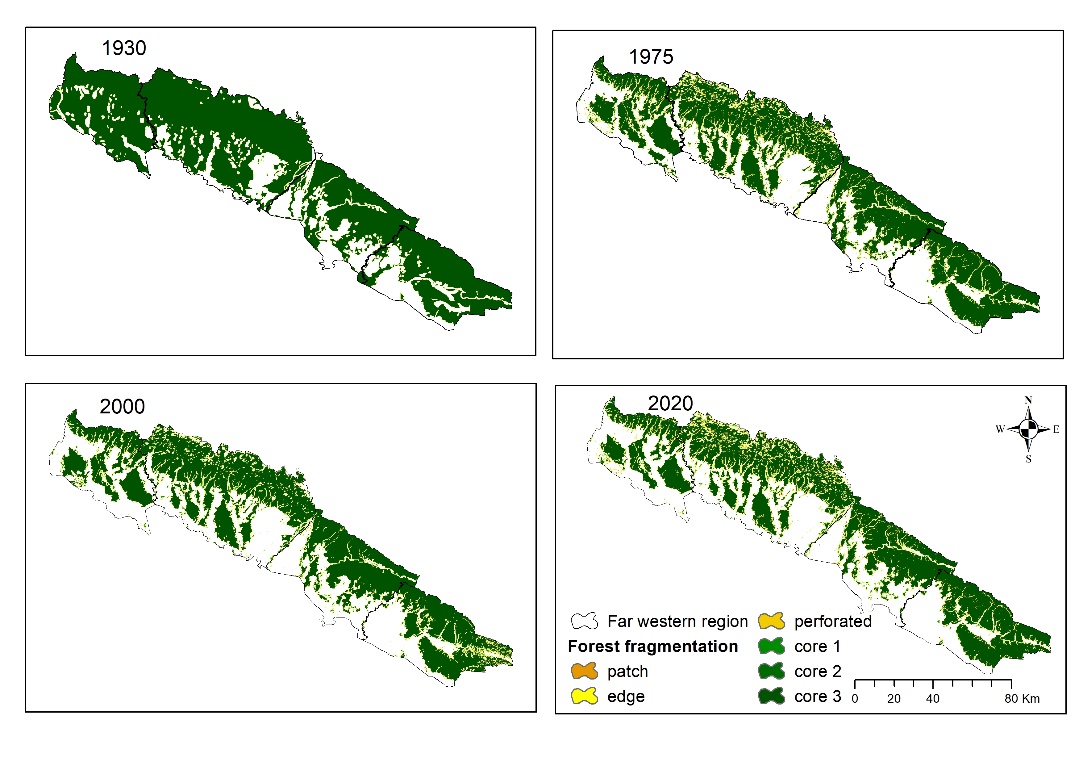


*Supplementary table S3.* Description of the terms used to quantify the forest fragmentation in Asian elephant habitat (Chure Terai Madhesh Landscape), Nepal.

| Metrics/variables | Code | Description |  |
| --- | --- | --- | --- |
| Mean Shape Index | MSI | Landscape shape index provides a simple measure of class aggregation or clumpiness. The LSI values range between 1 and infinity. The LSI value is 1 when the landscape consists of a single square or maximally compact (i.e., almost square) patch of the corresponding type and LSI increases without limit as the patch type becomes more disaggregated.  MSI equals the sum of the patch perimeter (m) divided by the square root of patch area  (m2) for each patch of the corresponding patch type, adjusted by a constant to adjust for a circular standard (vector) or square standard (raster), divided by the number of patches of the same type; in other words, MSI equals the average shape index (SHAPE) of patches ofthe corresponding patch type  . |  |
| Effective mesh size | MESH | The effective mesh size (MESH) measures forest fragmentation. The lower limit of MESH is constrained by the ratio of cell size to landscape area and is achieved when the corresponding patch type consists of a single one-pixel patch. MESH is maximum when the landscape consists of a single patch | (McGarigal et al., 2002) |
| Mean patch perimeter/area ratio | MPAR | It is shape complexity parameter describing the shape of different patches based on the relation between perimeter and area. It is used as a substitute for shape index. At class level, it decreases irregularly with the increase of area percentage for one class. |  |
| Patch |  | Patch pixels are within a small forest fragment that does not contain any core forest pixels | ^64^ |
| Edge |  | Assuming an edge width of 100 meters from the urban pixel, we consider edge. |  |
| Perforated and edge  (We assume edge width from the urban pixel as a edge and another 50 meter width as a perforated) |  | Perforated and edge forests are with 100 meters of urban pixels but are part of a tract containing core pixels:  Edge pixels are along the outside edge of the forest tract  Perforated pixels are along the edge of small forest gaps |  |
| core |  | core pixels are any forest pixels that are more than 100 meters from the nearest urban pixel |  |
| Core 1 |  | Core 1 or small core patches have an area of less than 250 acres (~1.1 km^2^) |  |
| Core 2 |  | Core 2 or medium core patches have an area between 250 and 500 acres (1.1-2.2 km^2^) |  |
| Core 3 |  | Core 3 or large core patches have an area greater than 500 acres (>2.2 km^2^) |  |

*Supplementary table S4.* Forest cover status in Nepal at 5x5 km^2^ grid cells.

1. Status of forest cover

| Class | 1930 | 1975 | 2000 | 2020 |
| --- | --- | --- | --- | --- |
| <1 km^2^ | 119 | 192 | 214 | 190 |
| 1-5 km^2^ | 182 | 174 | 166 | 168 |
| 5-10 km^2^ | 184 | 175 | 181 | 204 |
| 10-15 km^2^ | 189 | 253 | 264 | 289 |
| 15-20km^2^ | 262 | 335 | 347 | 334 |
| >20km^2^ | 656 | 456 | 384 | 332 |
|  | 1592 | 1585 | 1556 | 1517 |

1. Change in forest cover

| Deforestation | 1930-1975 | 1975-2000 | 2000-2020 | 1930 - 2020 |
| --- | --- | --- | --- | --- |
| <1 km^2^ | 343 | 1046 | 1196 |  |
| 1-5 km^2^ | 470 | 310 | 303 |  |
| 5-10 km^2^ | 215 | 39 | 4 |  |
| 10-15 km^2^ | 83 | 3 | 2 |  |
| 15-20km^2^ | 36 | 2 | 0 |  |
| >20km^2^ | 25 | 1 | 0 |  |
| 25km^2^ |  |  |  |  |
|  | 1172 | 1401 | 1505 |  |
| 1. Afforestation/ Restoration |  |  |  |  |
| <1 km^2^ | 215 | 181 | 51 |  |
| 1-5 km^2^ | 164 | 0 | 0 |  |
| 5-10 km^2^ | 74 | 0 | 0 |  |
| 10-15 km^2^ | 53 | 0 | 0 |  |
| 15-20km^2^ | 25 | 0 | 0 |  |
| >20km^2^ | 1 | 0 | 0 |  |
| Total | 532 | 181 | 51 |  |

Supplementary table S5. Change matrix of patch level forest fragmentation between 1930 and 2020.

| **1930/2020** | **Patch** | **Edge** | **Perforated** | **Core 1** | **Core 2** | **Core 3** | **Non-Forest** | **Total** |
| --- | --- | --- | --- | --- | --- | --- | --- | --- |
| **Patch** | 0 | 0.04 | 0.00 | 0.00 | 0.00 | 0.00 | 0.10 | **0.1** |
| **Edge** | 7.8 | 115.1 | 40.3 | 18.9 | 8.3 | 211.4 | 683.3 | **1085.1** |
| **Perforated** | 0.00 | 0.03 | 0.03 | 0.00 | 0.00 | 0.54 | 0.07 | **0.7** |
| **Core 1** | 0.18 | 3.06 | 1.35 | 1.00 | 0.20 | 5.11 | 31.03 | **41.9** |
| **Core 2** | 0.35 | 4.07 | 3.04 | 1.11 | 1.10 | 10.18 | 29.41 | **49.3** |
| **Core 3** | 81.9 | 2052.5 | 1282.0 | 248.1 | 112.4 | 12,012.1 | 7,347.4 | **23,136.5** |
| **Non-Forest** | 114.809 | 1106.032 | 360.054 | 238.785 | 86.283 | 947.325 | 15,289.132 | **18,142.42** |
| **Total** | **205.0** | **3280.9** | **1686.8** | **507.9** | **208.2** | **13186.6** | **23380.5** | **42456.0** |
